# Quantification and Progress Over Time of Specific Antibodies Against Severe Acute Respiratory Syndrome Coronavirus 2 in Breast Milk of Lactating Women Vaccinated With BNT162b2 Pfizer-BioNTech Coronavirus Disease 2019 Vaccine (LacCOVID)

**DOI:** 10.1093/ofid/ofac239

**Published:** 2022-05-11

**Authors:** Erika Esteve-Palau, Araceli Gonzalez-Cuevas, M Eugenia Guerrero, Clara Garcia-Terol, M Carmen Alvarez, Geneva Garcia, Encarna Moreno, Francisco Medina, David Casadevall, Vicens Diaz-Brito

**Affiliations:** Department of Infectious Diseases, Parc Sanitari Sant Joan de Deu, Sant Boi, Barcelona, Spain; Department of Microbiology, Parc Sanitari Sant Joan de Deu, Sant Boi, Barcelona, Spain; Department of Microbiology, Parc Sanitari Sant Joan de Deu, Sant Boi, Barcelona, Spain; Department of Obstetrics and Gynecology, Parc Sanitari Sant Joan de Deu, Sant Boi, Barcelona, Spain; Department of Infectious Diseases, Parc Sanitari Sant Joan de Deu, Sant Boi, Barcelona, Spain; Department of Infectious Diseases, Parc Sanitari Sant Joan de Deu, Sant Boi, Barcelona, Spain; Department of Infectious Diseases, Parc Sanitari Sant Joan de Deu, Sant Boi, Barcelona, Spain; Department of Infectious Diseases, Parc Sanitari Sant Joan de Deu, Sant Boi, Barcelona, Spain; Cancer Research Program, Hospital del Mar Research Institute, Barcelona, Spain; Department of Infectious Diseases, Parc Sanitari Sant Joan de Deu, Sant Boi, Barcelona, Spain

**Keywords:** breastfeeding, COVID-19, mRNA-based vaccination, passive immunity, SARS-CoV-2

## Abstract

**Background:**

Several observational studies demonstrated the passage of postvaccine antibodies through breast milk in women vaccinated against coronavirus disease 2019 (COVID-19), mostly with messenger RNA (mRNA)–based vaccines, but lacked long-term data.

**Methods:**

A 6-month prospective cohort study was performed to determine severe acute respiratory syndrome coronavirus 2 (SARS-CoV-2) vaccine–induced antibody levels in the breast milk of 33 lactating healthcare workers at different timepoints after mRNA BNT162b2 Pfizer-BioNTech COVID-19 vaccination. Moreover, we examined the correlation of SARS-CoV-2 antibody levels between serum and breast milk, adverse events related to vaccination, and rate of SARS-CoV-2 infections.

**Results:**

Mothers’ median age was 38 (interquartile range [IQR], 36–39) years and 15 (IQR, 10–22) months for infants. Median (IQR) SARS-CoV-2 immunoglobulin G (IgG) spike protein subunit S1 (S1) vaccine–induced levels at different timepoints for serum–milk pairs were 519 (234–937) to 1 (0–2.9) arbitrary units (AU)/mL at 2 weeks after first dose and 18 644 (9923–29 264) to 78 (33.7–128), 12 478 (6870–20 801) to 50.4 (24.3–104), 4094 (2413–8480) to 19.9 (10.8–51.9), 1350 (831–2298) to 8.9 (7.8–31.5) AU/mL at 2, 4, 12 and 24 weeks after second dose, respectively. We observed a positive correlation of antibody levels between serum and breast milk, no serious adverse events related to vaccination, and 2 (6%) COVID-19 vaccine breakthrough infections.

**Conclusions:**

Women vaccinated with Pfizer-BioNTech transmit antibodies into breast milk with a positive correlation with serum levels. Both decreased over time in a 6-month follow-up.

## Teaser

In this 6-month prospective cohort study, we observed the passage of BNT162b2 vaccine–induced antibodies to breast milk with a positive correlation with serum levels. Infants of breastfeeding vaccinated women could be acquiring vaccine antibodies for at least 6 months after vaccination.

Breastfeeding is one of the most efficacious means of preventing diseases and promoting health in both mothers and children [1]. Transfer of passive and active immunity through human milk is a key element in infant protection against infections [2, 3]. In the neonatal period, newborns are exposed to a myriad of microorganisms, whose main entry point is through mucosal barriers, and infants initially have an immune system that is too immature to cope with pathogens.

Breast milk contributes to a significant reduction in infant morbidity and mortality when breastfeeding is performed exclusively in the first 6 months of life [4–9]. Apart from its nutritional richness, both colostrum and mature milk have a high content of immunoglobulins, proteins, lactoferrin, and leukocytes, among other immunomodulatory factors, which makes it the first immunological contribution that the infant can receive in the first weeks and months of life [10, 11]. In addition to nonspecific immunological compounds, specific antibodies against different infections are transmitted through breast milk, acquired from the mother's previous contact with microorganisms or through the vaccines received against them [12, 13].

Severe acute respiratory syndrome coronavirus 2 (SARS-CoV-2) infection has an increased risk of severe adverse outcomes in unvaccinated people when comparing pregnant and nonpregnant women, particularly if infection occurs during the late second and early third trimesters. Moreover, SARS-CoV-2 infection in pregnant women is associated with an increased risk of preterm delivery, fetal growth restriction, stillbirth, and neonatal admission to the intensive care unit [14].

The coronavirus disease 2019 (COVID-19) pandemic has raised many questions among people who are breastfeeding, both because of the possibility of viral transmission to infants during breastfeeding and, with the approval of the SARS-CoV-2 vaccines, of the potential risks and benefits of vaccination in this specific population. Pregnant and breastfeeding women were excluded from all premarketing trials of COVID-19 vaccines, so some doubts exist regarding its compatibility. In this regard, a meta-analysis of 48 studies with 183 infected unvaccinated women analyzed the rate of SARS-CoV-2 genome identification in breast milk, concluding that this was found in 5% of cases, associated mainly with mild cases of COVID-19 in breastfed infants [15]. However, other studies have observed that although SARS-CoV-2 RNA was found through polymerase chain reaction (PCR) in the milk of infected women, these could not be detected in culture, suggesting that breast milk may not pose a risk of infection for the infant [16, 17]. Different studies during the pandemic suggest that, far from posing a risk of infection to the infant, breast milk from infected mothers may be protective as it contains specific antibodies against SARS-CoV-2 [15–18].

More recently, several observational studies have also demonstrated the passage of postvaccine antibodies through breast milk in women vaccinated against COVID-19, mostly with messenger RNA (mRNA)–based vaccines [19–23], but none showed long-term data. Thus, further research is needed to determine how long the antibodies are present in the breast milk of lactating mothers vaccinated against SARS-CoV-2.

Due to lack of knowledge in this field, our research group published the preliminary results in advance (1 month after mRNA vaccination), which demonstrated the passage of antibodies into breast milk [19]. Here we show the original research study with complete follow-up.

## METHODS

### Study Design, Endpoints, and Study Population

We conducted a prospective cohort study between February and September 2021 at Parc Sanitari Sant Joan de Déu, an urban medium-sized hospital in Spain covering an area of about 170 000 habitants, and carried out according to the Strengthening the Reporting of Observational Studies in Epidemiology (STROBE) reporting guidelines.

The primary endpoint was to determine SARS-CoV-2 vaccine–induced antibody levels in the breast milk of lactating women 4 weeks after mRNA BNT162b2 Pfizer-BioNTech COVID-19 complete vaccination (2 doses in 21 days). Secondary endpoints were to examine the correlation of SARS-CoV-2 antibody levels between serum and breast milk, analyze SARS-CoV-2 antibody levels (breast milk and serum) at different timepoints after vaccination, describe adverse events related to vaccination (AErVs) in both mothers and infants, and determine the rate of SARS-CoV-2 infections.

Inclusion criteria were lactating women >18 years of age who were vaccinated against SARS-CoV-2 with the BNT162b2 Pfizer-BioNTech COVID-19 vaccine during the vaccination campaign between January and March 2021. All were health workers.

Variables related to parity and breastfeeding were collected, as well as the medical history of both mothers and infants. The participants were also questioned at each visit about COVID-19–compatible symptoms in them or their infants and any adverse events related to the vaccine.

### Sample Collection

Serum and breast milk samples were simultaneously taken from each participant at different timepoints after vaccination: timepoint 1 (2 weeks after first dose), timepoint 2 (2 weeks after second dose), timepoint 3 (4 weeks after second dose), timepoint 4 (12 weeks after second dose), and timepoint 5 (24 weeks after second dose). Moreover, all participants underwent a COVID-19 antigen rapid test (Ag-RDT) at each timepoint.

### Antibodies and Ag-RDT Determination

When blood samples were obtained, they were centrifuged for 15 minutes at 3500 rpm and processed to determine levels of immunoglobulin G (IgG) antibodies against the spike protein subunit S1 (IgG-S1) and against the nucleocapsid (IgG-NC) of SARS-CoV-2 (Architect, Abbott). As vaccination does not induce nucleocapsid antibody response, any IgG-NC–positive result was considered a prior infection.

The milk samples were centrifuged for 15 minutes at 3500 rpm and after removing the fat layer with a pipette, the liquid layer of the milk was collected. Subsequently, we repeated the same process once more to determine IgG-S1 (Architect, Abbott). The determination was performed in milk only when obtaining positive IgG-NC in serum.

Nasopharyngeal swabs were analyzed with Panbio Abbot COVID-19 (Ag-RDT) to determine SARS-CoV-2 infection.

No tests were performed on the infants. In cases of suspicion of SARS-CoV-2 infection or close contact, the infants were referred to pediatrics to perform a PCR SARS-CoV-2 assay to exclude COVID-19.

### Statistical Analyses

Numeric variables were summarized using median and interquartile range (IQR, or Q1–Q3). Association between serum and milk SARS-CoV-2 antibodies was analyzed using repeated measures. Statistical analyses were performed with R version 4.0.3 (R Project for Statistical Computing), and figures were created using the ggplot2 R package.

## RESULTS

### Characteristics of Participants and Samples

A total of 33 volunteers were included in the study. The median age of mothers was 38 (IQR, 36–39) years and 15 (IQR, 10–22) months for the infants at the time of the vaccination. All mother and infant characteristics are described in Table 1.

**Table 1.** Participant Characteristics

Characteristic	No. (%) (N = 33)
Age, y, median (IQR)	38 (36–39)
Delivery type	
Natural vaginal delivery	21 (64)
Induced vaginal delivery–elective	9 (27)
Cesarean delivery	1 (3)
Emergency cesarean delivery	2 (6)
Gestational age (last delivery), wk, median (IQR)	40 (38–40)
Mother’s BMI, kg/m^2^, median (IQR)	21 (19.4–24.3)
Medical history	
*MTHFR* gene mutation	1 (3)
Early puberty	1 (3)
Intraepithelial neoplasia of cervix grade 3	1 (3)
Urethral stricture	1 (3)
Leiden factor V	1 (3)
Raynaud syndrome	1 (3)
Celiac disease	1 (3)
Autoimmune thyroiditis	1 (3)
Hypothyroidism	1 (3)
Tuberculosis in childhood	1 (3)
Infant sex	
Female	20 (60.6)
Male	13 (39.4)
Infant age, mo (at the time of the vaccine), median (IQR)	15 (10–22)
Birth weight, g, median (IQR)	3200 (3010–3570)
Breastfeeding	
Exclusive breastfeeding first 6 mo	32 (97)
Mixed breastfeeding	1 (3)
Profession	
Doctor	9 (27.3)
Physician	4
Surgeon	3
Psychiatrist	2
Nurse	7 (21.2)
Midwife	4 (12.1)
Nursing assistant	4 (12.1)
Social worker	3 (9.1)
Nutritionist	2 (6.1)
Administrative	2 (6.1)
Pharmacist	1 (3)
Laboratory technician	1 (3)

Data are presented as No. (%) unless otherwise indicated.

Abbreviations: BMI, body mass index; IQR, interquartile range; MTHFR, 5-methyltetrahydrofolate.

**Figure 1.**
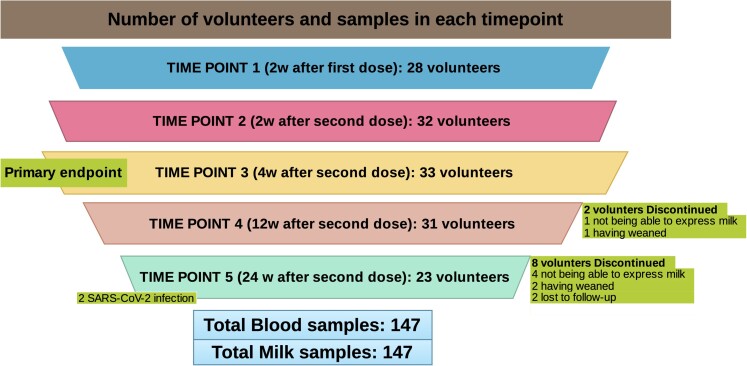
Number of volunteers and samples at each timepoint. Abbreviation: SARS-CoV-2, severe acute respiratory syndrome coronavirus 2.

We were able to recruit and obtain samples to determine the primary endpoint, antibody titers at 4 weeks after complete vaccination, in all participants (N = 33). However, we only achieved samples after the first dose of vaccine in 28 participants, and in 32 at 2 weeks after the second dose. In the 3-month follow-up, 2 participants were lost to follow-up (1 due to not being able to express milk, the other due to having weaned) and at 6-month follow-up a total of 8 participants were lost (4 were unable to express milk, 2 weaned, and 2 failed to visit). Number of volunteers and samples at each timepoint are shown in Figure 1.

No participants had confirmed SARS-CoV-2 prior infection at the beginning of the study (blood tests for IgG-NC and nasopharyngeal Ag-RDT were all negative). We collected and analyzed 147 serum and 147 milk samples from the volunteers.

Samples from timepoint 1 were taken at a median of 14 (IQR, 13–16) days after the first dose, while timepoints 2 and 3 samples were taken at 14 (IQR, 14–15) days and 28 (IQR, 28–30) days, respectively, after the second vaccine dose. Timepoint 4 samples were taken at 12-week follow-up (85 [IQR, 84–88] days) and timepoint 5 at 24-week follow-up (164 [IQR, 161–174] days).

### SARS-CoV-2 Vaccine–Induced Antibodies and Serum–Breast Milk Correlation

At timepoint 3 (4 weeks after second dose, primary endpoint), median IgG-S1 levels for serum–milk pairs were 12 478 (IQR, 6870–20 801) to 50.4 (IQR, 24.3–104) arbitrary units per milliliter (AU/mL). Results of all timepoints after vaccination are summarized in Table 2.

**Table 2.** Severe Acute Respiratory Syndrome Coronavirus 2 Vaccine–Induced Antibodies and Serum–Breast Milk Correlation

Sample	Timepoint 1: 2 Weeks After First Dose	Timepoint 2: 2 Weeks After Second Dose	Timepoint 3: 4 Weeks After Second Dose (Primary Endpoint)	Timepoint 4: 12 Weeks After Second Dose	Timepoint 5: 24 Weeks After Second Dose
IgG-S1 serum	519 (234–937)	18 644 (9923–29 264)	12 478 (6870–20 801)	4094 (2413–8480)	1288 (823–2233)
IgG-S1 breast milk	1 (0–2.9)	78 (33.7–128)	50.4 (24.3–104)	19.9 (10.8–51.9)	8 (4.9–19)

Data are presented as median (interquartile range) arbitrary units per milliliter (AU/mL).

Abbreviation: IgG, immunoglobulin G; S1, spike protein subunit S1.

Although maximum levels of IgG-S1, in both serum and milk, were observed 2 weeks after the second dose of vaccine with a subsequently progressive parallel decrease, we found an increase in antibody levels in 6-month follow-up samples in 2 participants related to a previous infection by SARS-CoV-2. In these 2 cases, a positive result of IgG-NC was also observed in serum but not in breast milk. Data of these patients were excluded for the timepoint 5 analysis.

**Figure 2.**
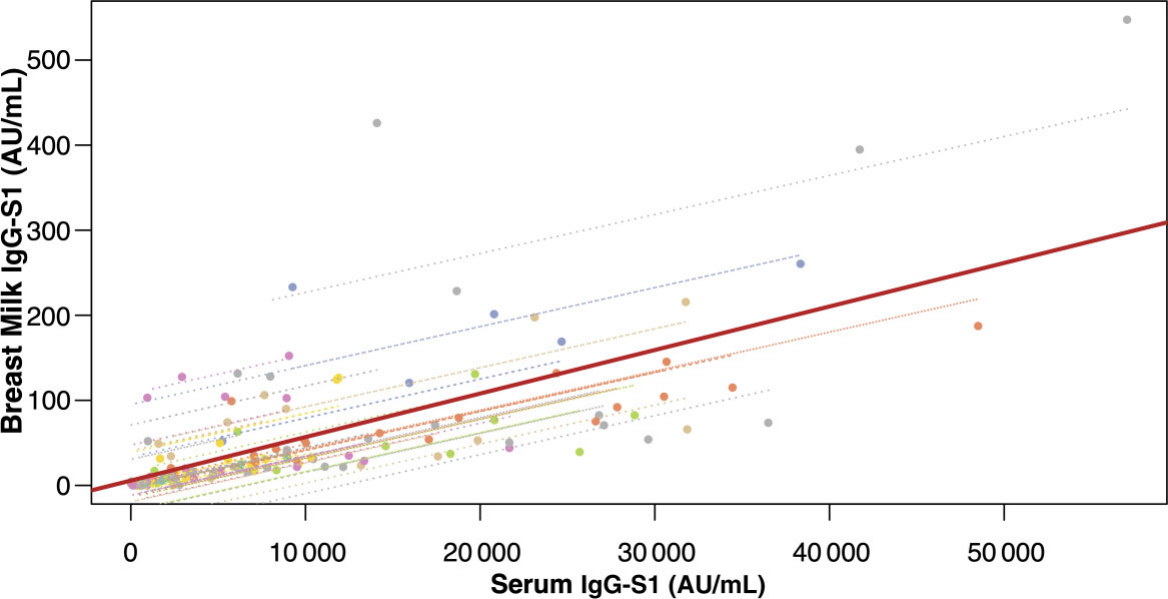
Correlation between immunoglobulin G spike protein subunit S1 (IgG-S1) levels (in arbitrary units [AU]/mL) in serum and breast milk of vaccinated participants. Correlation between serum and breast milk severe acute respiratory syndrome coronavirus 2 IgG-S1 levels overlaying patient-level regression lines. Interpatient variability, shown by the solid regression line, is parallel to intrapatient variability (dashed patient-level regression lines).

We examined the association between serum- and breast milk SARS-CoV-2 antibodies in 147 nonmissing data pairs from the 33 participants (113 degrees of freedom), obtaining a repeated measures (113) of 0.74 (95% confidence interval, .64–.81; *P* < 0.001) (Figure 2). These results suggest a moderate to strong positive correlation.

### Adverse Events Related to mRNA COVID-19 Vaccination

The main AErVs (mean of 2 doses) in our cohort were pain at the injection site (95%), feverish feeling and/or confirmed fever (15%), general malaise (15%), headache (11%), arthromyalgia (9%), asthenia (6%), axillary adenopathy (5%), and other (<3%). All AErVs are summarized in Table 3. No adverse events were observed in the infants. The number of accumulated AErVs or having systemic symptoms related to the vaccination did not correlate with an increased humoral immunogenicity.

**Table 3.** Vaccine-Related Adverse Events

Adverse Event	*First Dose^a^*	*Second Dose^b^*
*Local pain (mild-moderate)*	32 (97)	31 (94)
*Fever*	3 (9)	7 (21)
*General malaise*	3 (9)	7 (21)
*Headache*	2 (6)	5 (15)
*Arthromyalgia*	1 (3)	5 (15)
*Asthenia*	1 (3)	3 (9)
*Axillary adenopathy*	2 (6)	1 (3)
*Palmar erythema*	1 (3)	1 (3)
*Aphonia*	1 (3)	0
*Odynophagia*	0	1 (3)
*Nausea*	0	1 (3)
*Hypoglossal cyst*	0	1 (3)

Data are presented as No. (%). Total number of patients: N = 33.

a Vaccine-related adverse event (AErV) occurred during the 2 weeks after first-dose vaccination.

b AErV occurred during the 2 weeks after complete vaccination.

### SARS-CoV-2 Infections

During the study period, 2 participants had a COVID-19 vaccine breakthrough infection, 1 was completely asymptomatic, and the other had mild symptoms. In the first case, information on the contagion emerged from the analytical results of the 6-month follow-up, in which a rebound in the level of IgG-S1 was observed in serum and milk compared to the previous sample at timepoint 4 (from 10 448 AU/mL to 54 045 AU/mL, and 31 AU/mL to 121 AU/mL, respectively), as well as a positive result of the IgG-NC antibodies in serum with an index of 2.45, and a negative value in milk. In this case, both the mother and her infant were asymptomatic between 3 and 6 months of follow-up. No diagnostic test was performed at the time of infection as it was asymptomatic and no diagnosis was required.

The second case was diagnosed with COVID-19 a week before the 6-month follow-up, with symptoms of mild upper respiratory infection and fever. Once the quarantine period was completed, new samples were taken (12 days after diagnosis and after a negative control PCR), showing a considerable increase in IgG-S1 in serum and milk compared to the previous determination at timepoint 4 (from 2611 AU/mL to 14 425 AU/mL, and 17 AU/mL to 73 AU/mL, respectively) and an IgG-NC in serum with an index of 1.02 and negative in milk. The baby had fever and mild upper respiratory symptoms a few days later but no diagnostic test was performed since the mother had an obvious diagnosis and refused to perform a test on her child.

**Figure 3.**
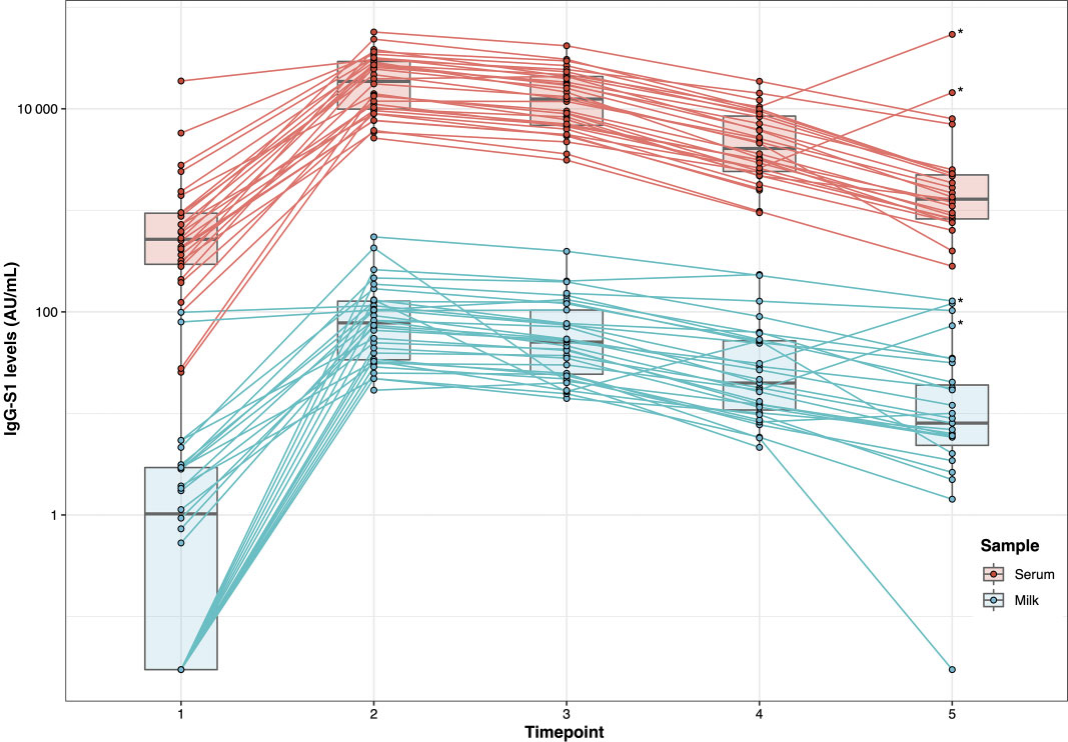
Progress over time of immunoglobulin G spike protein subunit S1 (IgG-S1) levels (in arbitrary units [AU]/mL) in breast milk and serum of vaccinated participants. Timepoint (TP) 1: 2 weeks after first dose; TP2: 2 weeks after second dose; TP3: 4 weeks after second dose; TP4: 12 weeks after second dose; TP4: 24 weeks after second dose. *Two patients were excluded for the 6-month analysis (TP5) because they suffered a severe acute respiratory syndrome coronavirus 2 infection.

These 2 patients were excluded for the 6-month analysis (timepoint 5). However, their values are included in Figure 3.

## DISCUSSION

To our knowledge, this is one of the few studies conducted with a 6-month follow-up [24]. In the 3- and 6-month follow-up, we observed a progressive decrease in antibody levels in serum samples, which correlates with data from previous studies carried out in healthcare professionals vaccinated with the BNT162b2 vaccine [25, 26]. Additionally, in our study, we observed that this decrease in serum antibody levels is associated with a parallel decrease in breast milk levels at 3 and 6 months, with the same serum–breast milk positive correlation. Therefore, it is reasonable to hypothesize that serum determination of SARS-CoV-2 IgG-S1 could indicate approximately the breastmilk levels of antibodies during the 6 months after vaccination.

Duration of transplacental immune protection against SARS-CoV-2 in infants of mRNA-vaccinated women is unknown due to lack of follow-up data, but could probably protect the infant for at least the neonatal period [27]. As our data show, despite late decrease antibody levels in breast milk, infants of breastfeeding vaccinated women could be acquiring vaccine antibodies for at least 6 months after vaccination. However, despite observing the passage of antibodies into breast milk, their protective efficacy against COVID-19 in infants and the cutoff in breast milk for anti–SARS-CoV-2 protection remain unknown.

The current COVID-19 pandemic has raised multiple concerns for breastfeeding mothers. At first, due to the potential risk of infecting babies, breastfeeding was even contraindicated in infected women. After the approval of the SARS-CoV-2 vaccines, fears regarding breastfeeding centered on the potential harmful effects that these could have on babies. For this reason, and in the absence of scientific evidence, vaccination in lactating women was initially contraindicated in many settings, often leaving the choice of vaccination and/or maintaining breastfeeding in the hands of mothers.

Pregnant and breastfeeding women are often excluded from clinical trials with new drugs due to the fears and ethical dilemmas that these entail. This means that the safety of most drugs (including vaccines) is principally evaluated in postmarketing monitoring.

Given the lack of evidence regarding vaccination against COVID-19 in early 2021, Parc Sanitari Sant Joan de Déu promoted the LacCOVID study, which included lactating women who carry out their work on the front line and who decided to get vaccinated at the beginning of the vaccination campaign while continuing to breastfeed.

During the pandemic, different studies have shown that the infective virus is rarely transmitted through the milk of infected mothers [15–17] and, instead, specific antibodies against the virus are transmitted to the babies through breast milk [19–23]. The passage of IgG-specific antibodies into breast milk is also confirmed in our study in vaccinated women; in addition, we were able to verify how these levels have a correlation with those observed in serum. These results are consistent with those observed in other recent research [22, 23]. To date, in addition to our manuscript, there is only 1 study conducting long-term follow-up on the transmission of these antibodies to breast milk [24].

Our participants at the time of vaccination had infants with a median age of 15 (IQR, 10–22) months at the time of enrollment. This population differs from previous studies, where infants were postpartum or not more than 6 months of age, and in this sense, our study shows new data in this field. Human milk after the second year of lactation contains significantly higher concentrations of IgG compared with the first months of age; this fact supports prolonged lactation to protect children's health. However, the IgG values in our study may therefore not be reflective of IgG values in breast milk of women vaccinated immediately postpartum [28].

In our cohort, we observed 2 cases of vaccine breakthrough infections in the 6-month follow-up (6%), both in the final study period (between 3 and 6 months). Although there are limited data on the incidence of COVID-19 in pregnant women, there are no studies in the literature on SARS-CoV-2 risk of infection in breastfeeding women. Further research is needed to estimate COVID-19 incidence in this group.

We have also observed how, in cases in which a participant became infected by SARS-CoV-2, the levels of IgG-S1 showed a new increase in both serum and breast milk. Not so in the case of IgG-NC which, despite having been positive in serum, did not have its presence in milk confirmed. A possible explanation could be that IgG-NC is a qualitative technique, and the limit of detection could be higher than IG-S1 quantitative antibody determination.

On the basis of the substantial increase in IgG-S1 in serum and milk observed after SARS-CoV-2 infection in 2 volunteers, it may be reasonable to consider that a COVID-19 vaccine booster shot could increase serum and milk levels in the same way as a natural infection.

The AErVs observed in our cohort are similar to those demonstrated by the clinical trial conducted for the approval of the vaccine [26].

The main limitation of this study is its small sample size. In our sample, there was only 1 infant of exclusive breastfeeding age (<6 months), so it was not possible to analyze whether there is a greater passage of antibodies to milk in this period compared to a longer postpartum period. Moreover, with a larger sample, we could have confirmed the correlation between antibody levels in serum and breast milk that we found and predict the levels in milk from a serum sample determination.

Another limitation is the lack of prevaccine antibody levels. However, we carried out serial levels of IgG against the spike protein and against nucleocapsid, to discern whether any participant had had the infection in the previous months, and no volunteers had IG-NC–positive results after 2 weeks of first immunization. We did not perform neutralization studies on the isolated antibodies, which may be considered a limitation. Nevertheless, a recent publication showed that IgG antibodies in human milk after mRNA vaccination was associated with an IgG-dominant response that exhibited a neutralization activity against live SARS-CoV-2 virus, so its effectiveness could be assumed in our study [29]. A further limitation is the lower sensitivity of Ag-RDT compared to the PCR SARS-CoV-2 assay; it is possible that some asymptomatic cases were missed. Last, the use of different techniques for the analysis of the 2 types of IgG antibodies (IgG-NC and IgG-S1) and lack of immunoglobulin A and RBD antibody determination, in addition to the fact that Abbott Architect does not have an emergency use authorization for antibody measurements in milk, could lead to a bias in the interpretation of the data.

Most of the studies in pregnant and lactating women to date have been conducted after vaccination with mRNA vaccines. This could be a limitation, so more studies with other vaccines are needed to be able to extrapolate these data to other types of vaccine.

Finally, larger prospective studies are needed to confirm the safety of SARS-CoV-2 vaccination in individuals who are breastfeeding and further assess the association between vaccination and infants’ health and SARS-CoV-2–specific immunity.

## CONCLUSIONS

The results of this study show that women vaccinated with Pfizer-BioNTech transmit antibodies into breast milk and, in addition, these levels correlate with those observed in serum. IgG serum levels induced by the vaccine decrease over time in a 6-month follow-up, as do the antibodies in breast milk. However, despite late decrease antibody levels in breast milk, infants of breastfeeding vaccinated women could be acquiring vaccine antibodies for at least 6 months after vaccination.

Vaccination against SARS-CoV-2 has been shown to drastically reduce cases of serious infections, as well as the transmissibility of the virus. In breastfeeding mothers, this strategy may protect the babies, both by generating group immunity and thus reducing the risk of contracting the infection in their social environment, as well as by the passage of antibodies through breast milk.

## Notes

***Author contributions***. Concept and design: E. E.-P. and V. D.-B. Recruitment of volunteers and collection of laboratory samples: M. C. A., C. G.-T., E. E.-P., G. G., E. M., and F. M. Analysis of laboratory samples: A. G.-C. and M. E. G. Acquisition, analysis, or interpretation of data: All authors. Drafting of the manuscript: E. E.-P., D. C., and V. D-.B. Statistical analysis: E. E.-P. and D. C. Supervision: V. D-.B. Critical revision of the manuscript for important intellectual content: All authors. V. D.-B. and E. E.-P. had full access to all of the data in the study and take responsibility for the integrity of the data and the accuracy of the data analysis.

***Financial support***. This work had not financial support.

***Patient consent statement***. The study was explained to all volunteers in detail, and all gave written informed consent prior to participating. The Sant Joan de Déu Research Foundation local ethics committee approved this study.

***Potential conflicts of interest***. All authors report no potential conflicts of interest.
